# The aryl hydrocarbon receptor and retinoid receptors cross-talk at the *CYP1A1* promoter *in vitro*

**DOI:** 10.17179/excli2018-1147

**Published:** 2018-03-15

**Authors:** Stefanie Hessel-Pras, Anke Ehlers, Albert Braeuning, Alfonso Lampen

**Affiliations:** 1 German Federal Institute for Risk Assessment, Department Food Safety, Max-Dohrn-Str. 8-10, 10589 Berlin, Germany

**Keywords:** cytochrome P450, dioxin, drug metabolism, enzyme induction, RXR

## Abstract

The epithelium of the small intestine plays an important role in detoxification processes due to the presence of various xenobiotic-metabolizing enzymes from phase I and II, as well as transport proteins of the ATP-binding cassette superfamily. Exposure to xenobiotics induces the expression of these proteins in the small intestine, with multiple signaling pathways stimulated by exogenous compounds converging at individual gene promoters by mechanisms which have not been fully understood yet. In this context the promoter region of the *CYP1A1* gene, encoding the phase I monooxygenase cytochrome P450 1A1, was analyzed by chromatin immunoprecipitation with regard to binding of xeno-sensing receptors following stimulation of Caco-2 cells with agonists of the aryl hydrocarbon receptor (AHR) and retinoid receptors. Histone acetylation in the regulatory region of *CYP1A1* was enhanced by treatment with 2,3,7,8-tetrachlorodibenzo-p-dioxin (TCDD) or all-*trans* retinoic acid (at-RA). Binding of retinoid-X-receptor (RXR) α to the promoter region was detected in response to at-RA, while AHR bound to the gene promoter following its activation by TCDD. Of note, enhanced RXRα binding was also detected after AHR stimulation, and increased AHR binding was observed after retinoid receptor activation by at-RA. Exposure of Caco-2 cells to mixtures of AHR and retinoid receptor agonists yielded synergistic induction of *CYP1A1* mRNA. In conclusion, the present data improve our knowledge on retinoic acid-dependent effects on *CYP1A1* expression and demonstrate unexpected mixture effects by cross-talk of the different receptors.

## Introduction

The epithelium of the small intestine is the first physical and biochemical barrier for compounds or contaminants which are ingested via the food. Resorbed small hydrophilic xenobiotics are often excreted chemically unchanged via the bile or urine, whereas lipophilic substances have to be metabolized into excretable hydrophilic metabolites or conjugates. Phase I of this biotransformation process comprises oxidation or reduction reactions, and frequently polar functional groups such as hydroxyl (-OH), thiol (-SH), or amine (-NH_2_) groups are introduced into a target molecule. These reactions are primarily catalyzed by cytochrome P450 monooxygenases (CYP). Some phase I metabolites require further modifications during phase II of xenobiotic metabolism, where metabolites are conjugated with endogenous hydrophilic molecules (for example glutathione, sulfate, or glucuronic acid) by enzymes such as glutathione *S*-transferases, sulfotransferases or UDP-glucuronosyltransferases. The elimination of phase I and/or phase II metabolites as well as unchanged drugs is mediated by an active transport accomplished by transport proteins belonging to the ATP-binding cassette (ABC) superfamily. For a review of the role of the intestinal epithelium in xenobiotic metabolism, please refer to the review by Kaminsky and Zhang (2003[13]). 

Many aspects of *in vivo* intestinal metabolism can be resembled *in vitro* by use of the human Caco-2 cell model (Buesen et al., 2002[4], 2003[3]; Ebert et al., 2005[5]; Münzel et al., 1999[21]; Tamura et al., 2001[30]). After differentiation this tumor-derived cell line develops typical morphological characteristics of the polarized human small intestinal epithelium and expresses a wide variety of xenobiotic-metabolizing enzymes, as well as proteins of the ABC transporter superfamily (Boulenc et al., 1992[2]; Buesen et al., 2002[4]; Meinl et al., 2008[20]; Münzel et al., 1999[21]; Peters et al., 1989[24]; Scharmach et al., 2009[28]; Tamura et al., 2001[30]).

Exposure by xenobiotics can modulate the expression of xenobiotic-metabolizing enzymes in the small intestine by various mechanisms, not all of which have been fully understood so far. Nuclear or cytosolic receptors which act as ligand-activated transcription factors upon binding of xenobiotics play a key role in the transcriptional regulation of genes related to drug metabolism, such as the aryl hydrocarbon receptor (AHR, also termed dioxin receptor), the constitutive androstane receptor, or the pregnane-X-receptor. Nuclear receptors are responsible for complex signaling cross-talk and pharmacokinetic interactions; for review see e.g. Plant and Aoubadi (2009[25]). While several cellular consequences of activation of individual nuclear receptors have been analyzed in-depth, there are still knowledge gaps with regards to the actions exerted by other receptor types such as for example the retinoid receptors. Elucidation of the regulation of drug metabolism under real-life conditions is furthermore complicated by the fact that exposure actually occurs not to single chemical entities, but rather to complex mixtures of a multitude of compounds acting via different molecular modes of action, e.g. by activating different nuclear receptors which interact with respect to target gene regulation. 

The *CYP1A1* gene, encoding the phase I enzyme cytochrome P450 1A1, is regulated by the AHR and widely recognized as model target gene of this receptor, which is activated by planar aromatic hydrocarbon, dioxins, and other chemically related compounds (Kawajiri and Fujii-Kuriyama, 2007[14]). Binding of the AHR to the *CYP1A1* promoter has been subject to extensive research, e.g. see Kress et al. (1998[16]). In addition, previous studies have demonstrated an influence of retinoids on *CYP1A1* gene expression (Fallone et al., 2004[7]; Gambone et al., 2002[9]; Jurima-Romet et al., 1997[12]; Li et al., 1995[18]; Ohno et al., 2012[23]; Vecchini et al., 1995[32]; Wanner et al., 1995[33]). The presence of a retinoic acid response element (RARE) in the *CYP1A1* promoter region has also been demonstrated (Vecchini et al., 1994[31]). 

The aim of this study was therefore to further analyze the transcriptional regulation of *CYP1A1* in the human adenocarcinoma cell line Caco-2 by agonists of the AHR and the different retinoic acid receptors, alone or in combinations. It was hypothesized that receptor crosstalk may be a key mechanism mediating the occurrence of unexpected mixture effects which might deviate from simple addition of the effects caused by exposure to an individual compound activating only one single type of receptor. The obtained data help in gaining a better understanding of the regulation of xenobiotic detoxification by individual nuclear receptor agonists and their mixtures.

## Material and Methods

### Chemicals

All-*trans* retinoic acid (at-RA) and 2,3,7,8-tetrachlorodibenzo-*p*-dioxin (TCDD) were purchased from Sigma-Aldrich (Taufkirchen, Germany). The RAR agonist Am580 (CD336; [4-(5,6,7,8-tetrahydro-5,5,8,8,-tetramethyl-2-naphthalenylcarboxamido)benzoic acid]) and the RXR agonist CD2608 (LG1069; (4-[1-(4,5,5,8,8-pentamethyl-5,6, 7,8-tetrahydro-2-naphthyl)-ethyl] benzoic acid) were kindly provided by Dr. U. Reichert (CIRD-Galderma, Sophia-Antipolis, France). All other chemicals were purchased from Merck (Darmstadt, Germany) or Sigma-Aldrich in the highest available purity.

### Cell culture

The human colon adenocarcinoma cell line Caco-2 (ECACC, Porton Down, UK) was cultured in Dulbecco's modified Eagle's medium (DMEM; PAA Laboratories GmbH, Coelbe, Germany) supplemented with 10 % fetal calf serum, 100 IU/ml penicillin, and 100 µg/ml streptomycin at 37 °C in a humidified atmosphere of 5 % CO_2_. Cells from passages 29-40 were used for all experiments. Caco-2 cells were cultured in six-well plates until complete confluency and were further cultivated for 14 to 18 days in order to allow differentiation. For a recent detailed description of Caco-2 cell culture, please refer to Lichtenstein et al. (2017[19]). Verification of successful differentiation into enterocyte-like cells, which was also used as a verification of cell line identity for Caco-2 cells, has also been described in detail in the aforementioned previous publication (Lichtenstein et al., 2017[19]).

### Gene expression analysis

Cells were treated with test compounds in concentrations as indicated in the figures. Compounds were always freshly dissolved in culture medium prior to treatment, from stock solutions prepared in dimethyl sulfoxide (DMSO). The final DMSO concentration in the treatment medium was 0.1 % (v/v). After 24 h of incubation, the medium was removed, the cell monolayer was washed with ice-cold phosphate-buffered saline (PBS), and cells were harvested for subsequent RNA isolation. All compounds were used at non-cytotoxic concentrations, as determined by routine cytotoxicity testing, to ensure the absence of unspecific effects caused by cellular stress.

### Preparation of RNA and real-time quantitative PCR analysis

Total RNA was isolated using the RNeasy kit (Qiagen, Hilden, Germany) according to the manufacturer's protocol including a DNase digestion step. The concentration and quality of the RNA was determined with a Nano Drop spectrophotometer (Nanodrop Technologies, Wilmington, DE, USA). 2 µg of RNA were reverse transcribed by 200 U Moloney murine leukemia virus reverse transcriptase (Promega, Heidelberg, Germany) using 100 pmol of oligo (dT)_15_ as primers (2 h at 42 °C). Real-time quantitative PCR was performed on a 7900HT real-time PCR cycler (Applied Biosystems, Darmstadt, Germany). Experiments were set up in triplicates containing SYBR Green Mastermix (AbGene, Hamburg, Germany) and the respective primers (3 pmol per reaction). The primer sequences are available from Table 1(Tab. 1). The thermal cycling protocol comprised an initial denaturation step at 95 °C for 15 min, followed by 40 cycles of denaturation at 95 °C for 15 s, annealing and extension at 60 °C for 1 min, and a final extension at 60 °C for 15 min. The mRNA amount of *CYP1A1* in each sample was normalized to its *ACTB* (encoding β-actin) content and then referred to vehicle controls treated with the solvent DMSO only to obtain fold inductions.

### Chromatin immunoprecipitation

For chromatin immunoprecipitation (ChIP) experiments differentiated Caco-2 cells grown in 175 cm² cell culture flasks were used. Cells were treated for 1.5 h with TCDD, at-RA, or vehicle (DMSO), respectively. ChIP analyses were performed as described in detail by Ehlers et al. (2008[6]), where a more detailed description of the experimental protocol can be found. Immunoprecipitation was carried out with antibodies directed against acetyl-histone H3 (H3-K9/K14, 06-599; Upstate, Temecula, CA, USA), RXRα (D 20), RXRβ (C 20), and AHR (H 211) (all from Santa Cruz Biotechnology, Santa Cruz, CA, USA). 

**Figure 1:**
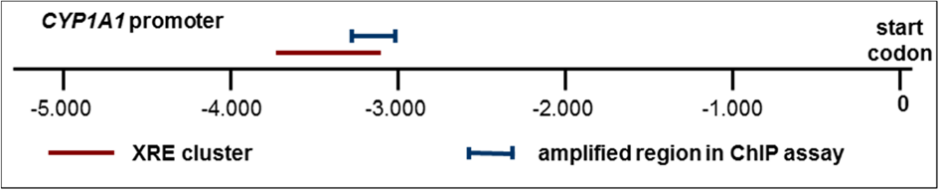
Schematic delineation of the *CYP1A1* promoter with the XRE cluster containing known AHR binding sites, and with the genomic region used for amplification in the ChIP assays.

A genomic region around 3,000 base pairs upstream of the transcription starting point of *CYP1A1* was chosen for amplification following ChIP. This region corresponds to the XRE cluster (i.e., a region containing multiple AHR-binding so-called xenobiotic response elements) in the *CYP1A1* promoter (Figure 1(Fig. 1)). Subsequent to the digestion of RNA and proteins, the enriched fragmented DNA was analyzed for the abundance of the promoter fragments of interest, in comparison to the input control as well as control samples from solvent-treated cells. Successful enrichment of immunoprecipitated DNA was demonstrated by real-time quantitative PCR via comparison with the amount of DNA in the input control. In addition, the second exon of the *PAX5* gene located more than 13,000 base pairs downstream of its start codon was amplified as a negative control to demonstrate specificity of DNA enrichment. The DNA content of each sample was normalized to its *ACTB* content and then referred to input DNA to obtain fold enrichment. Primer sequences are shown in Table 1(Tab. 1).

### Statistical analysis 

Statistical analyses were performed with SigmaPlot software. Statistical significance of differences between mean values were determined by Student's t-test or, for concentration-dependent studies, by one-way ANOVA followed by Dunnett's post-hoc test. Statistically significant differences were assumed at p < 0.05.

## Results

### Induction of CYP1A1 gene expression by AHR, RAR and RXR agonists

**Figure 2:**
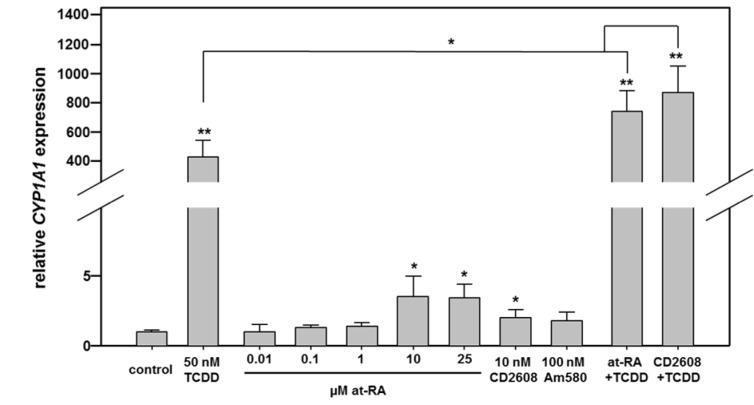
Real-time RT-PCR analysis of *CYP1A1* mRNA expression in Caco-2 cells following stimulation with different nuclear receptor agonists. Cells were treated with agonists of the AHR, the different retinoid receptors, or combinations of both. Combination of RXRα and AHR activation yields over-additive effects. Mean + SD (n ≥ 3 independent experiments) are shown. Statistical significance is indicated as follows: *, p < 0.05; **, p < 0.01. For detailed data from individual replicates, please refer to the Supplementary Data Table.

First, Caco-2 cells were exposed to agonists of different nuclear receptors to characterize the impact of activation of these signaling pathways on *CYP1A1* mRNA expression (Figure 2(Fig. 2); for detailed data from individual replicates, please refer to the Supplementary Data Table). The real-time PCR data show that the model AHR agonist TCDD, as expected, drastically stimulated *CYP1A1* gene expression. Exposure of cells to 50 nM TCDD induced *CYP1A1* gene expression by approximately 425-fold (Figure 2(Fig. 2)). Activation of RAR was initially accomplished by incubation of cells with increasing concentrations of at-RA. Only at concentrations of 10 µM or higher, a slight, but statistically significant approximately 3.5-fold increase in *CYP1A1* expression was recorded, while lower concentrations of at-RA did not exert remarkable effects (Figure 2(Fig. 2)). Activation of RAR with 100 nM of the agonist Am580 did not significantly affect *CYP1A1* expression, whereas activation of RXR by its ligand CD2608 led to a very slight, but significant approximately 2-fold increase in *CYP1A1* mRNA (Figure 2(Fig. 2)).

### Histone H3 acetylation status of the CYP1A1 promoter

Both, the AHR and the retinoid receptors, act as ligand-dependent transcription factors which bind to the promoters of target genes and thereby regulate their transcription. This is generally associated with an increased histone acetylation of the respective promoter region, and with increased binding of the activated transcription factors to their recognition sequences within the gene regulatory region of the DNA. The histone H3 acetylation status of the promoter of the *CYP1A1* gene was therefore analyzed by chromatin immunoprecipitation (ChIP) assays (see also scheme in Figure 1(Fig. 1)). Differentiated Caco-2 cells were exposed to 50 nM TCDD for AHR activation or to 10 µM at-RA for RAR activation. Immunoprecipitations from nuclear cellular Caco-2 extracts were carried out with an antibody against acetylated histone H3 and a DNA region approximately 3,000 base pairs upstream of the transcription start was amplified from the precipitates by PCR. 

**Figure 3:**
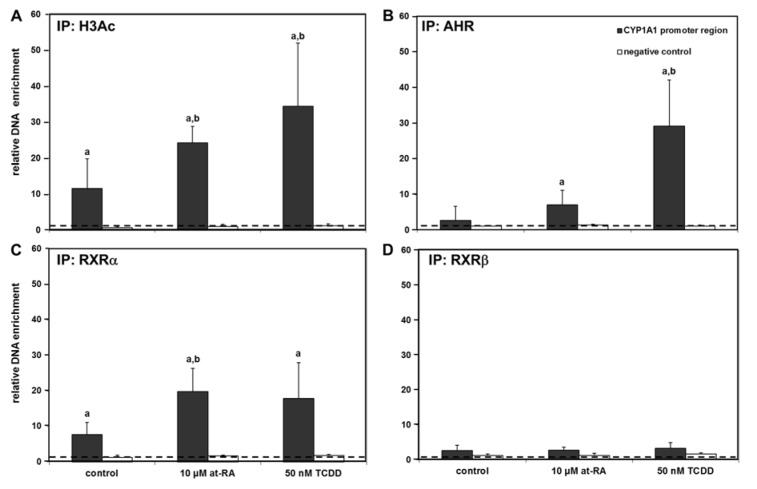
Chromatin immunoprecipitation at the *CYP1A1* promoter. Immunoprecipitation results with antibodies directed against acetylated histone H3 (H3Ac) (A), the AHR (B), RXRα (C), and RXRβ (D) are presented. The dashed line indicates the level at which no enrichment of the respective promoter region is present. Negative controls (amplification of the *PAX5* coding region not responsive to the AHR or retinoid receptors) are shown as white bars for comparison. Mean + SD (n ≥ 4 independent experiments) are shown. Statistical significance is indicated as follows: ^a^, p < 0.05 vs. input control; ^b^, p < 0.05 vs. solvent control (i.e., treatment effect). For detailed data from individual replicates, please refer to the Supplementary Data Table.

Figure 3A(Fig. 3) demonstrates the histone H3 acetylation status in the promoter region of *CYP1A1* (-3,000 bp upstream of the start codon) in Caco-2 cells (for detailed data from individual replicates, please refer to the Supplementary Data Table). In control cells only treated with the solvent, histone H3 showed some constitutive acetylation in the promoter region of *CYP1A1* even without xenobiotic treatment, while a substantial enhancement in the amplified DNA amount, corresponding to an increased acetylation of histone H3 in the respective genetic region, was recorded following treatment with both, at-RA and TCDD (Figure 3A(Fig. 3)). Histone H3 acetylation was not detected in the negative control, where a DNA region outside of any promoter region (i.e., the coding region of the gene *PAX5*; Figure 3A(Fig. 3)) was amplified; this demonstrates the specificity of our assay. 

### AHR, RXRα and RXRβ binding to the CYP1A1 gene promoter

Increased histone H3 acetylation in the promoter region of *CYP1A1* following xenobiotic treatment was observed (Figure 3A(Fig. 3)). This was plausible since histone acetylation is generally associated with gene activation. We now analyzed whether also the recruitment of the respective relevant transcription factors, namely AHR and retinoic acid receptors, to the same regions of the gene promoters would be detectable by ChIP assays. Activation of the AHR again was achieved by treatment with TCDD, while RAR activation was performed using at-RA. ChIP analyses were carried out with antibodies directed against the AHR, RXRα and RXRβ. In control cells treated with the solvent only, AHR and RXRβ were barely associated with the promoter of the *CYP1A1* gene, whereas basal binding significantly differing from non-antibody controls was detected for RXRα (Figure 3(Fig. 3)). The known interaction between the AHR and the *CYP1A1* promoter (-3,000 base pairs) was verified in samples from cells exposed to TCDD which showed massive binding of the AHR to this genetic region (Figure 3B(Fig. 3)). In addition, an increase in RXRα binding to the *CYP1A1* promoter was also observed following treatment of Caco-2 cells with TCDD (Figure 3C(Fig. 3)). No major impact of TCDD treatment was observed with regard to the binding of RXRβ (Figure 2D(Fig. 2)). Cell treatment with at-RA resulted in a statistically significant enrichment of the RXRα-*CYP1A1* promoter interaction (Figure 3C(Fig. 3)), without affecting RXRβ binding to the gene promoter (Figure 3D(Fig. 3)). Interestingly, an increase in AHR binding to the *CYP1A1* promoter was also visible following cell treatment with at-RA (Figure 3B(Fig. 3)). Taken together these results indicate that individual treatment with either at-RA or TCDD might, to a certain degree, be able to assist the recruitment of both receptors, AHR and RXRα, to the *CYP1A1* gene promoter, even without addition of an exogenous ligand of the second receptor.

Experiments with at-RA concentrations in a range from 0.1 µM to 25 µM verified the generated data for the histone H3 acetylation at the *CYP1A1* promoter and demonstrated the increased binding of AHR and RXRα (Table 2(Tab. 2); for detailed data from individual replicates, please refer to the Supplementary Data Table). Moreover, substantial enrichment was now also seen for RXRβ, but only at the highest tested concentration of at-RA (Table 2(Tab. 2)). For histone H3 acetylation and AHR binding, lower concentrations of at-RA caused higher enrichment at the *CYP1A1* promoter (Table 2(Tab. 2)). Enrichment was again not detected in the negative control in these experiments (data not shown).

### AHR and retinoid receptor agonist mixture effects on CYP1A1 expression

The data presented above indicate a cross-talk between the different receptors. Recently it has been shown that the cross-talk of the AHR with Wnt/β-catenin signaling at the *CYP1A1* promoter synergistically enhances the responses to AHR agonists (Schulthess et al., 2015[29]). We were now interested whether similar effects might also be observed with AHR agonists and retinoids activating their respective receptors. Thus, in subsequent mixture experiments, the RXR agonists CD2608 and at-RA were combined with TCDD (Figure 2(Fig. 2); right bars). The stimulation of both pathways, namely activation of the AHR with TCDD combined with an activation of the RAR/RXR-dependent signaling pathways by at-RA or CD2608, enhanced *CYP1A1* mRNA expression by up to 870-fold, which is significantly more than expected from the same concentration of the AHR agonist alone, and also significantly more than expected when additive effects of stimulation of the two individual signaling pathways is assumed. Thus a combined stimulation of AHR- as well as RAR/RXR-dependent transcription enhances the cellular levels of *CYP1A1* mRNA in an over-additive manner (Figure 2(Fig. 2)).

## Discussion

The connection between *CYP1A1* expression and xenobiotic activation of the AHR has long been known and constitutes a rather well-investigated example of the transcriptional regulation of a drug-metabolizing enzyme by xenobiotics via a ligand-activated receptor. However, additional factors which modulate *CYP1A1* expression independently of the AHR and/or interact with AHR-dependent signaling in the regulation of the *CYP1A1* gene are much less understood (see also INTRODUCTION section and references therein).

Here, we incubated Caco-2 cells with agonists of the AHR and different retinoid receptors. Our data demonstrate a pronounced induction following AHR activation. This corresponds well to the known inducibility of the gene by AHR agonists, e.g. see Kimura et al. (1986[15]) and many further publications demonstrating that the *CYP1A1* gene is regulated via the AHR signaling pathway upon activation of the receptor by compounds such as for example TCDD or 3-methylcholanthrene (Fernandez-Salguero et al., 1996[8]; Prasch et al., 2003[26]). With regards to the *CYP1A1* promoter, basal constitutive acetylation, along with pronounced hyper-acetylation upon exposure of the cells to TCDD was observed. This goes well with data by Nakajima et al. (2003[22]), who reported that the inhibition of histone deacetylases or of DNA methylation causes an increase in gene expression of *CYP1A1* in HeLa cells. 

Data regarding the consequences of retinoid receptor activation on *CYP1A1* expression are conflicting: it has been reported that at-RA either down-regulates (Li et al., 1995[18]; Wanner et al., 1995[33]) or up-regulates (Vecchini et al., 1995[32]) *CYP1A1* gene expression in keratinocytes. In hepatocytes, at-RA had a weak inducing effect on *CYP1A1* mRNA expression (Jurima-Romet et al., 1997[12]), whereas opposing observations have been made in HepG2 hepatocarcinoma cells (Ohno et al., 2012[23]; Fallone et al., 2004[7]). We now detected clearly inductive effects of at-RA on *CYP1A1* in human intestinal Caco-2 cells. The way by which retinoid receptors affect the *CYP1A1* gene promoter thus possibly differs between different cell types and/or is affected by further variables such as the chosen cell culture conditions. Nonetheless, CYP1A1-inducing effects of at-RA, if observed in an *in vitro* system, seem to be generally much less pronounced than the corresponding effects exerted by a potent AHR agonist. It should be noted in this context that at-RA is primarily an activator of RAR, not RXR. Nonetheless, it has been shown that at-RA binding to the RAR is the most important ligand needed for the activation of the RAR-RXR transcription factor heterodimer; e.g. see the review by Blomhoff and Blomhoff (2006[1]). This warrants the analysis of RXR binding to the DNA in response to at-RA treatment.

Interactions between the AHR and RAR/RXR have been described in the context of physiological organism development, but not for xenobiotic metabolism-related functions of the receptors: for example, an important cross-talk occurs during the development of the medaka fish (Hayashida et al., 2004[11]), where at-RA induces *AhR* gene expression which is necessary for the development of blood vessels and bones in the fish embryos. Additionally, it was observed that rodent *Cyp1a1* expression varies during cell differentiation (Reiners et al., 1992[27]) and developmental stages (Giachelli et al., 1991[10]), two processes strongly influenced by retinoid-dependent signaling. In our study the treatment of Caco-2 cells with TCDD resulted in the expected substantial increased binding of the AHR to the *CYP1A1* promoter region, which contains known XREs (Kubota et al., 1991[17]; Kress et al., 1998[16]). Of note, at-RA alone also caused an increased binding of the AHR to the *CYP1A1* promoter. Vice versa, AHR activation by TCDD caused also binding of RXRα to the promoter, as did RAR activation by at-RA. Data on transcription factor binding are corroborated by at-RA- and TCDD-induced hyperacetylation of the gene promoter. The present findings from ChIP analyses suggest a cross-talk between the receptors at the *CYP1A1* promoter. A step further at the level of transcription, a very interesting observation is that treatment of Caco-2 cells with a mixture of the AHR agonist TCDD and different retinoid receptor agonists resulted in a strongly over-additive effect on *CYP1A1* mRNA expression. This unexpected synergistic mixture effect points towards a substantial cross-talk of the different receptors at the gene promoter or via their downstream signaling events. The *CYP1A1* promoter is known to by subject to synergistic activation processes, either by multiple AHR proteins binding to individual, cooperatively-acting binding sites in the gene promoter, or by different transcription factors which also allow for synergistic effects in case both are activated by upstream signaling. Cooperativity at multiple AHR binding sites as well as the synergistic behavior of AHR- and β-catenin-dependent signaling have recently been dissected in depth at the gene promoter level (Schulthess et al., 2015[29]). The present data now point towards the existence of similar cross-talk mechanisms between the AHR and retinoid receptors. In fact, interactions between the AHR and RAR/RXR signaling pathway have been described previously: Gambone et al. (2002[9]) showed that certain synthetic retinoids are also activating ligands of the AHR. Moreover, a retinoic acid response element (RARE) has been identified in the *CYP1A1* promoter (Vecchini et al., 1994[31]), while the authors of the latter paper did not broach the issue of cross-talk between the different receptors. Of note, the RARE identified by Vecchini et al. (1994[31]) is located approximately 1000 base pairs upstream of the transcription start of the first exon of *CYP1A1* and therefore not identical to the RXRα-interacting genomic region recognized in our analyis (see scheme in Figure 1(Fig. 1)). 

The crosstalk of AHR and RXRα surely warrants deeper investigation in follow-up studies. These will help revealing the AHR/RXRα cross-talk in more detail, and will further add to our knowledge on the regulatory network influencing the activity of the AHR. The present data constitute an instructive example on how multiple exogenous compounds are capable of influencing cellular targets in a synergistic manner, thus questioning if a general assumption of a maximally additive behavior of individual foreign compounds in toxicology sufficiently protects from unwanted effects.

## Acknowledgements

This work was supported by the German Research Foundation (DFG) (grant LA 1177/4-2).

## Conflict of interest disclosure

The authors declare that they have no conflict of interest.

## Supplementary Material

Supplementary data

## Figures and Tables

**Table 1:**
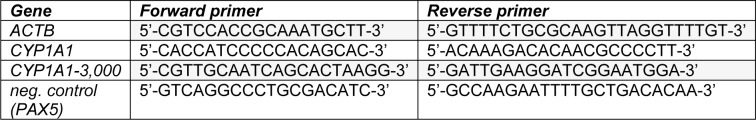
Primer sequences used for real-time quantitative PCR

**Table 2:**
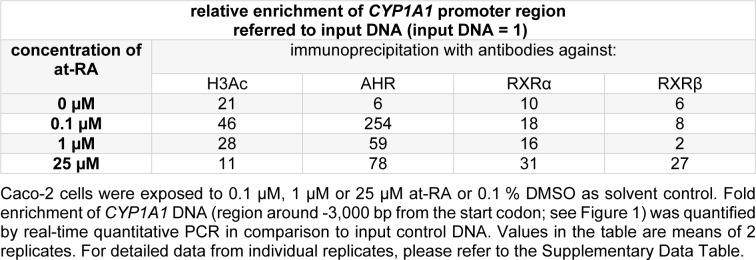
ChIP analysis of *CYP1A1* promoter binding of H3Ac, AHR, RXRα and RXRβ
