# Supplementary material for: The aryl hydrocarbon receptor and retinoid receptors cross-talk at the CYP1A1 promoter in vitro
Source: EXCLI J. 2018 Mar 15;17:246–56. doi: 10.17179/excli2018-1147 (PMC5938535; doi:10.17179/excli2018-1147)
Supplement: Supplementary data [file EXCLI-17-246-s-001.pdf]

EXCLI Journal 2018;17:246-256 – ISSN 1611-2156
Received: February 19, 2018, accepted: March 08, 2018, published: March 15, 2018

Supplementary data to:

Original article:

THE ARYL HYDROCARBON RECEPTOR AND RETINOID RECEPTORS CROSS-TALK AT THE *CYP1A1* PROMOTER *IN VITRO*

Stefanie Hessel-Pras, Anke Ehlers, Albert Braeuning*, Alfonso Lampen

German Federal Institute for Risk Assessment, Department Food Safety,
Max-Dohrn-Str. 8-10, 10589 Berlin, Germany

* Corresponding author: Albert Braeuning, German Federal Institute for Risk Assessment, Department Food Safety, 10589 Berlin, Germany; phone +49-30-18412-3758; fax +49-30-18412-63758; e-mail: albert.braeuning@bfr.bund.de

http://dx.doi.org/10.17179/excli2018-1147

This is an Open Access article distributed under the terms of the Creative Commons Attribution License (http://creativecommons.org/licenses/by/4.0/).

EXCLI Journal 2018;17:246-256 – ISSN 1611-2156
Received: February 19, 2018, accepted: March 08, 2018, published: March 15, 2018

Supplementary Table 1: Summary Data_IP *CYP1A1*

x-fold enrichment

IP	*primer*	*treatment*	input	replicate 1	replicate 2	replicate 3	replicate 4	replicate 5	mean	SD
**H3Ac**	*CYP1A1-3000*	*control*	**1.0**	4.6	3.1	12.4	23.7	13.9	**11.6**	8.3
		10 µM at-RA	**1.0**	30.9	21.6	21.2	23.7		**24.3**	4.5
		50 nM TCDD	**1.0**	9.4	25.1	38.0	47.1	52.9	**34.5**	17.5
	PAX-5	*control*	**1.0**	0.7	1.0	0.9	0.4	0.3	**0.7**	0.3
		10 µM at-RA	**1.1**	0.7	0.4	1.9	1.5	0.7	**1.1**	0.6
		50 nM TCDD	**1.0**	1.4	0.7	2.0	1.2	0.7	**1.2**	0.6
**AHR**	*CYP1A1-3000*	*control*	**0.9**	0.7	0.5	1.3	8.6		**2.8**	3.9
		10 µM at-RA	**1.2**	7.4	5.2	12.7	3.4		**7.2**	4.0
		50 nM TCDD	**1.0**	16.6	32.1	46.1	22.1		**29.2**	12.9
	PAX-5	*control*	**0.8**	1.2	1.2	1.2	1.0		**1.2**	0.1
		10 µM at-RA	**1.0**	1.8	1.3	1.3	1.4		**1.4**	0.3
		50 nM TCDD	**1.0**	0.7	1.3	1.5	1.2		**1.2**	0.3
**RXRalpha**	*CYP1A1-3000*	*control*	**1.0**	6.4	3.3	10.4	10.5		**7.7**	3.5
		10 µM at-RA	**1.0**	29.1	17.0	18.9	14.0		**19.8**	6.5
		50 nM TCDD	**1.0**	16.4	32.5	10.5	11.9		**17.8**	10.1
	PAX-5	*control*	**1.1**	0.9	0.8	2.1	1.4		**1.3**	0.6
		10 µM at-RA	**1.0**	1.7	2.0	1.5	1.4		**1.7**	0.2
		50 nM TCDD	**1.0**	1.9	1.5	1.5	2.1		**1.7**	0.3
**RXRbeta**	*CYP1A1-3000*	*control*	**1.1**	3.0	1.0	3.9			**2.6**	1.5
		10 µM at-RA	**1.0**	2.8	1.8	3.5			**2.7**	0.9
		50 nM TCDD	**1.0**	1.4	3.4	4.8			**3.2**	1.7
	PAX-5	*control*	**1.0**	1.5	0.7	1.4			**1.2**	0.4
		10 µM at-RA	**1.0**	1.9	1.1	0.5			**1.2**	0.7
		50 nM TCDD	**1.1**	2.0	1.3	1.7			**1.7**	0.3

EXCLI Journal 2018;17:246-256 – ISSN 1611-2156
Received: February 19, 2018, accepted: March 08, 2018, published: March 15, 2018

Supplementary Table 2: Concentration dependency atRA

promoter region	**treatment**	***input***		***H3Ac***		***input***		***AhR***	
		replicate 1	replicate 2	replicate 1	replicate 2	replicate 1	replicate 2	replicate 1	replicate 2
CYP1A1 -3.000	*control*	1.1	0.9	23.3	18.2	1.0	1.0	3.6	8.2
	0,1 µM at-RA	1.0	1.0	49.6	42.3	0.9	1.1	206.5	302.2
	1 µM at-RA	1.0	1.1	28.9	26.1	0.0	0.1	48.9	68.3
	25 µM at-RA	1.0	1.0	10.0	12.8	1.4	0.7	77.8	78.0
PAX-5	*control*	0.9	1.1	1.5	0.6	0.7	1.4	2.3	3.6
	0,1 µM at-RA	1.5	2.0	0.7	1.3	0.5	1.9	1.7	3.0
	1 µM at-RA	0.7	1.4	1.6	1.0	0.6	1.7	2.8	3.6
	25 µM at-RA	1.0	1.0	1.4	1.2	0.8	1.3	3.0	3.5
promoter region	**treatment**	***input***		***RXRalpha***		***input***		***RXRbeta***	
		replicate 1	replicate 2	replicate 1	replicate 2	replicate 1	replicate 2	replicate 1	replicate 2
CYP1A1 -3.000	*control*	1.2	0.8	10.4	10.5	1.4	0.7	6.6	6.1
	0,1 µM at-RA	0.9	1.1	18.1	16.7	1.1	0.9	9.7	6.8
	1 µM at-RA	1.0	1.0	16.4	14.8	1.5	0.6	2.0	1.6
	25 µM at-RA	1.1	0.9	24.0	36.8	1.3	0.8	26.9	27.1
PAX-5	*control*	0.9	1.1	3.7	3.4	0.7	1.4	0.8	1.2
	0,1 µM at-RA	0.6	1.7	2.2	1.4	0.3	0.8	2.6	0.7
	1 µM at-RA	1.3	0.8	2.4	2.6	0.6	1.6	3.6	1.5
	25 µM at-RA	1.1	0.9	3.5	1.1	0.8	1.3	1.5	1.2

EXCLI Journal 2018;17:246-256 – ISSN 1611-2156
Received: February 19, 2018, accepted: March 08, 2018, published: March 15, 2018

Supplementary Table 3: Induction of gene expression *CYP1A1*

treatment	**1. replicate**	**2. replicate**	**3. replicate**	**mean**	**standard deviation**
control	1.0	1.2	0.8	1.0	0.17
50 nM TCDD	297.9	523.4	454.4	425.3	115.56
0,01 µM at-RA	1.6	0.5	0.9	1.0	0.54
0,1 µM at-RA	1.4	1.4	1.1	1.3	0.17
1 µM at-RA	1.6	1.5	1.1	1.4	0.29
10 µM at-RA	2.6	2.7	5.3	3.5	1.50
25 µM at-RA	4.6	3.0	2.9	3.5	0.99
10 nM CD2608	2.0	2.6	1.5	2.0	0.60
100 nM Am580	1.6	1.4	2.5	1.8	0.60
1 µM at-RA + 50 nM TCDD	593.3	750.1	871.7	738.4	139.55
10 nM CD2608 + 50 nM TCDD	828.4	1.066.6	710.0	868.3	181.61
